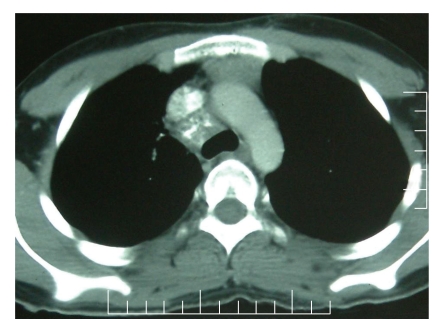# Anterior Mediastinal Mass: A Rare Presentation of Tuberculosis

**DOI:** 10.1155/2011/635385

**Published:** 2011-03-07

**Authors:** Gopi C. Khilnani, Neetu Jain, Vijay Hadda, Sudheer K. Arava

**Affiliations:** ^1^Department of Medicine, All India Institute of Medical Sciences, New Delhi 110 028, India; ^2^Department of Pathology, All India Institute of Medical Sciences, New Delhi 110 028, India

## Abstract

We report a case of a 14-year-boy who presented to us with a low-grade fever with evening rise for 9 months. Along with this, the patient also reported a reduction in his appetite and body weight. He had a mild dry cough but no respiratory symptoms otherwise. There was no other localization for fever on history. He received antitubercular therapy, based on abnormal chest radiograph. However, there was no relief in his symptoms. General physical examination revealed mild fever. Systemic examination was unremarkable. Blood investigations done for fever were noncontributory. Computed tomographic (CT) scan of the chest revealed a mediastinal mass compressing the trachea. The possibilities of lymphoma or germ cell tumour were considered. A biopsy from the mass under CT guidance was performed. The histopathology revealed multiple epithelioid cell granulomas with necrosis, and the diagnosis of tuberculosis was made. The clinical course of this patient and the relevant literature is presented in this paper.

## 1. Introduction

The anterior mediastinal space is almost a virtual space. However, the multiplicity of the structures it contains and the diversity of disease processes affecting them make it a region of great clinical interest. This cavity encloses muscle, ligaments, fat tissue, and parenchymatous organs such as thymus, thyroid, and ectopic parathyroid gland. Mediastinal masses are commonly encountered in clinical practice. However, they represent a challenging and urgent diagnostic problem because the differential diagnoses range from absolutely benign to highly malignant conditions, and delay in diagnosis may be fatal. The common anterior masses include thymoma, lymphoma, and germ cell tumours [1, 2]. An isolated mediastinal mass without lung parenchymal lesion is an uncommon presentation of tuberculosis [3, 4]. Here, we present a case of anterior mediastinal mass compressing the trachea which was histopathologically diagnosed as tubercular and treated successfully with antituberculosis therapy (ATT).

## 2. Case Summary

A 14-year-old boy presented to us in July 2009 with low-grade, evening rise fever since March 2008. He also complained of reduction in appetite and weight loss of 8 kg during this period. He had a mild dry cough. However, there was no history of shortness of breath, chest pain, hemoptysis, hoarseness of voice, or dysphagia. There was no other significant history to localize the cause of fever. He received antituberculosis therapy (ATT) for these symptoms and abnormal chest radiograph. He was treated for the first time for 4 months from March to June 2008 and then for 6 months in a private hospital without any relief in his symptoms. 

### 2.1. Physical Examination

On examination, the patient was febrile with an oral temperature of 37.3°C. His pulse (88/minute), blood pressure (126/88 mmHg), and respiratory rate (14/minute) were normal. His body weight was 39 kg. There was no peripheral lymph node enlargement. There was no facial puffiness or neck vein prominence. The examination of chest, cardiovascular, abdomen, and nervous system was unremarkable. There was no testicular enlargement.

### 2.2. Laboratory and Radiographic Findings

**Figure 1:**
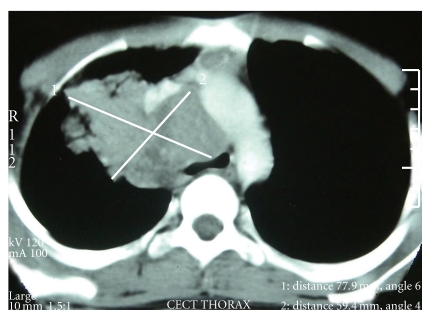
CT scan of the chest is showing a homogenous mass measuring 7.5 cm × 6.0 cm. The mass is compressing the trachea causing significant lumen compromise.

Blood investigations revealed a normal hemoglobin (12.3 gm/dL), total and differential leukocyte counts (7000/dL; neutrophils: 65%, lymphocytes: 33%, monocytes: 1%, and eosinophils: 1%), and platelets (230 × 10^3^/dL). The erythrocyte sedimentation rate was 43 mm during the 1st hour. The results of clinical biochemistry including renal and hepatic functions were normal. ELISA for HIV was negative. Chest radiograph revealed a mediastinal mass. A contrast-enhanced computed tomograph (CECT) scan of chest was done for further characterization of the mass. It showed a homogenous anterior mediastinal mass compressing the trachea (Figure 1). Based on the clinical and radiological features, possibilities of lymphoma or a germ cell tumour were considered.

### 2.3. Clinical Course

**Figure 2:**
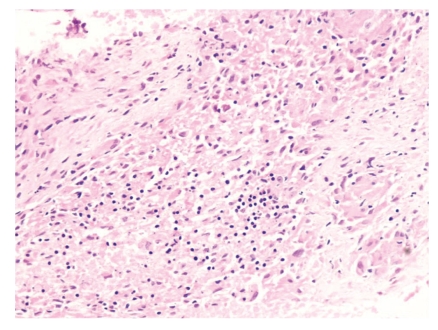
Photomicrograph showing epithelioid cell granulomas with necrosis (H & E stain; 20x).

At another centre, fine needle aspiration cytology (FNAC) done from the mass showed few ill-defined granulomas. Culture and drug sensitivity for *Mycobacterium tuberculosis* could not be done due to lack of this facility at that centre. Based on FNAC report, he received ATT twice without any relief. Therefore, he was referred to us for further management. In view of his young age, compression of trachea, and involvement of vessels by the mass, we considered a possibility of lymphoma and germ cell tumour. Tuberculosis, despite high prevalence in this country, was not a strong consideration because of unusual presentation and lack of response to ATT. For histopathological diagnosis, CT-guided cutting needle biopsy from the mass was done. It revealed ill-formed epithelioid cell granulomas with necrosis (Figure 2). Stain for acid fast bacillus was negative. Biopsy sample was sent for culture and drug sensitivity which was reported to be negative at the end of six weeks. 

Based on the presence of necrotizing granulomas, a diagnosis of tuberculosis was entertained. Considering the possibility of drug resistance, based on the past history of treatment with antituberculosis drugs twice, a 6-drug ATT regimen that included rifampicin (450 mg), isoniazid (300 mg), ethambutol (800 mg), pyrazinamide (1250 mg), streptomycin (0.75 gm), and levofloxacin (750 mg) along with pyridoxine was started. He showed improvement in symptoms after two months and radiological improvement on chest radiograph done after three months. A follow-up CECT scan of the chest done after 9 months of ATT showed almost complete resolution of the mass (Figure 3). The ATT was reduced to two drugs including isoniazid with pyridoxine and rifampicin with an intention to give it for 3 more months.

## 3. Discussion

Tuberculosis can have protean manifestations but presenting as an isolated mediastinal mass without parenchymal lesion is unusual [3, 5]. It is, however, not so unusual in immune-compromised patients [6]. Usual differential diagnoses of an anterior mediastinal mass include thymoma, lymphoma, teratomatous neoplasms, thyroid mass, vascular masses, lymph node enlargement due to metastases or granulomatous disease, and pleuropericardial and bronchogenic cysts. Among these, thymoma is more common in the elderly. However, in younger patients like ours, lymphoma and germ cell tumours are more likely [1]. It may be very difficult to differentiate radiologically one from the other. Certain features on CT scan such as attenuation values suggesting fat, water, calcium, or necrosis may help in narrowing down the differential diagnoses. In addition, the exact location and morphology of the mass along with clinical features such as patient age, gender, signs and symptoms, and laboratory values, may further help in narrowing the list of possible aetiologies. These all help in directing appropriate additional imaging or other diagnostic procedure/s or therapeutic approaches [1, 2, 7]. However, the definite diagnosis requires histopathological examination of the tissue from the mass [1, 2, 8]. On histopathology, both thymoma and lymphoma are round cell tumours and it is almost impossible to differentiate both on microscopy alone. However, there are certain immune-histochemical markers which can help in achieving the final diagnosis. Non-Hodgkin lymphoma and Hodgkin lymphoma can be differentiated from thymoma by positive staining for CD45, CD20, CD15, and CD30, respectively [9]. Cytologic and immunocytochemical features which help in making the diagnosis of thymic carcinoma are clear-cut cytological atypia, absence of immature lymphocytes (CD1a+ and CD99+), and expression of CD5 and CD70 by neoplastic epithelial cells [9]. Mediastinal seminomas are immunoreactive for placental alkaline phosphatase (PLAP) and CD117, while CD30 is expressed in 85–100% of embryonal carcinoma [9]. Germ cell tumours may be diagnosed based on histopathology and associated increase in serum *β*-hCG or *α*-fetoprotein levels. In our patient, histopathology revealed epithelioid cell granulomas with necrosis. The granulomatous lesion may be due to sarcoidosis, tuberculosis, or fungal infections. Sarcoidosis is characterized by nonnecrotizing granulomatous inflammation. Both tubercular as well as fungal granulomas may be necrotic. In order to differentiate between them, Ziehl-Neelsen staining is a quick and definitive method of diagnosis. However, it is not so frequently positive. 

A striking feature in this patient was involvement of vessels and narrowing of trachea by the mass, which is unusual with tubercular lymphadenopathy. This presentation is more common with teratomas, specifically malignant teratomas or lymphoma, both of which are common during adolescence [7]. Germ cell tumours are predominately cystic in nature, although they frequently have a soft tissue component, often with a thin outer capsule. Fat and calcium are also common elements. The mass in our patient was solid without any fat or calcium. A malignant teratoma can present as a solid mass. It is frequently associated with serological markers such as *α*-fetoprotein and *β*-human chorionic gonadotrophin. In our case, both were negative. Finally, CT-guided biopsy from the mediastinal mass achieved the diagnosis. The presence of epithelioid cell granulomas with necrosis and complete resolution of the mass with ATT confirmed our diagnosis of tuberculosis. The patient, however, did not respond to ATT on two previous occasions. When he was started on ATT for the first time, he developed drug-induced hepatitis and hence was non-compliant and not only stopped the treatment before completing full course but also missed several days of treatment in between. The next time, he was advised ATT from DOTS in view of his poor compliance, but he declined. During this period, his compliance to ATT was good. The poor response to treatment might be due to drug-resistant tuberculosis. Incidence of multidrug-resistant tuberculosis in previously treated patient is reported to be 8–67% in India [10]. In view of the possibility of drug-resistant tuberculosis, a biopsy specimen was sent for culture and drug sensitivity testing and the patient was started on six drugs, two of which were not used previously. We planned to modify treatment as per the dug sensitivity report which was inconclusive. As the patient showed clinical and radiological improvement, he was continued on the same regimen.

In conclusion, tubercular infection can have varied presentations. Though presentation as an isolated mediastinal mass is rare in immunocompetent adults, with the worldwide resurgence of tuberculosis owing to the “AIDS epidemic” and the growing number of drug-resistant tuberculosis strains, the importance of suspecting this condition in the appropriate clinical setting cannot be overemphasized. With prompt commencement of ATT, it is possible to treat these patients successfully.

## 4. Summary

Our case presented with low-grade fever without any localization on history and physical examination. Radiology revealed an anterior mediastinal mass compressing the trachea. As described in the literature, the final diagnosis could only be confirmed after histopathological examination. However, the diagnosis of tuberculosis was not strongly suspected. Once diagnosed, tuberculosis in most of the cases may be treated easily. In our case, however, the patient had received ATT prior to coming to us without any relief in symptoms. Therefore, he was treated with augmented ATT instead of 4 drugs. With this treatment, the patient showed clinical as well as radiological response. Therefore, the possibility of multidrug-resistant TB cannot be ruled out. However, the lesson from this case is that TB can closely mimic malignant lesions on radiology but can be treated with better outcome compared to other common mediastinal masses. Therefore, it should always be considered among differential diagnoses of mediastinal mass.

**What Is Known on This Topic?**

Tuberculosis can have varied presentations and can involve any part of the body. Anterior mediastinal masses are usually thymoma, lymphoma, and germ cell tumours.

**What Does This Add?**

Being a treatable condition with good outcome, it should be considered as one of the differential diagnoses of mediastinal masses. This is very important in view of rising incidence of tuberculosis with ongoing AIDS epidemic.

##  Declaration

The paper is the authors' own original work and has not been published or is not being considered for publication elsewhere.

## Figures and Tables

**Figure 3:**